# Correlation of pre-existing comorbidities with disease severity in individuals infected with SARS-COV-2 virus

**DOI:** 10.1186/s12889-024-18457-2

**Published:** 2024-04-15

**Authors:** Jasmina Marušić, Edhem Hasković, Adnan Mujezinović, Vedran Đido

**Affiliations:** 1 Department of Health Care, Faculty of Medicine, University of Zenica, Zenica, Bosnia and Herzegovina; 2 Department of Biology, Faculty of Science, University of Sarajevo, Sarajevo, Bosnia and Herzegovina; 3 Department of Public Health, Faculty of Health Studies, University of Sarajevo, Sarajevo, Bosnia and Herzegovina; 4 Marjanovića put 39, 72000 Zenica, Bosnia and Herzegovina

**Keywords:** Comorbidities, Cardiovascular diseases, Hypertension, Diabetes mellitus, Urinary system diseases

## Abstract

Shortly after the first publication on the new disease called Coronavirus Disease 2019 (Covid-19), studies on the causal consequences of this disease began to emerge, initially focusing only on transmission methods, and later on its consequences analyzed in terms of gender, age, and the presence of comorbidities. The aim of our research is to determine which comorbidities have the greatest negative impact on the worsening of the disease, namely which comorbidities indicate a predisposition to severe Covid-19, and to understand the gender and age representation of participants and comorbidities. The results of our study show that the dominant gender is male at 54.4% and the age of 65 and older. The most common comorbidities are arterial hypertension, diabetes mellitus, and cardiovascular diseases. The dominant group is recovered participants aged 65 and older, with comorbidities most frequently present in this group. The highest correlation between patients with different severity of the disease was found with cardiovascular diseases, while the coefficient is slightly lower for the relationship between patients with different disease severity and urinary system diseases and hypertension. According to the regression analysis results, we showed that urinary system diseases have the greatest negative impact on the worsening of Covid-19, with the tested coefficient b being statistically significant as it is 0.030 < 0.05. An increase in cardiovascular diseases affects the worsening of Covid-19, with the tested coefficient b being statistically significant as it is 0.030 < 0.05. When it comes to arterial hypertension, it has a small impact on the worsening of Covid-19, but its tested coefficient b is not statistically significant as it is 0.169 > 0.05. The same applies to diabetes mellitus, which also has a small impact on the worsening of Covid-19, but its tested coefficient b is not statistically significant as it is 0.336 > 0.05. Our study has shown that comorbidities such as urinary system diseases and cardiovascular diseases tend to have a negative impact on Covid-19, leading to a poor outcome resulting in death, while diabetes mellitus and hypertension have an impact but without statistical significance.

## Introduction

The consequences of infection with the Severe Acute Respiratory Syndrome Coronavirus 2 (SARS-CoV-2) virus, which has infected over 530 million people worldwide, are still being investigated, especially for individuals with comorbidities [1]. Diagnosis of Covid-19 in individuals with comorbidities requires a detailed medical history, physical examination, as well as analysis of laboratory and radiological data [2]. Some discrepancies may be the result of false positivity in COVID-19 testing, and a positive/negative result of a COVID-19 test is simply a test result. More parameters need to be taken into consideration before evaluating a possible COVID-19 case, and these are the parameters that influence studies like this one [3]. These steps are important to differentiate Covid-19 symptoms from symptoms of other diseases associated with comorbidities, as well as to assess the severity of the infection and tailor therapy accordingly. It is important to determine whether a comorbidity represents an independent risk or whether it is mediated by other factors such as age or gender [1, 2]. The Centers for Disease Control and Prevention (CDC) summarize comorbidities that have a significant association with an increased risk of severe COVID-19 illness. The list is continuously updated, and understanding which condition increases the risk and their relative significance for adverse outcomes is still evolving [4]. Previous studies suggest that risks generally increase with age and are higher in men, but there is also strong evidence showing increased risks in individuals with various comorbidities; including cardiovascular diseases, diabetes mellitus, chronic kidney disease, liver and lung diseases, obesity, immunodeficiencies, certain mental state disabilities [5]. In line with relevant reports and prior research, it has been observed that cardiovascular diseases are more common in patients diagnosed with COVID-19. This raises important inquiries concerning the heightened susceptibility of individuals with cardiovascular comorbidities to the novel coronavirus. Furthermore, it prompts a closer examination into the impact of hypertension and existing cardiovascular conditions on the advancement of the illness, as well as the predicted prognosis and outcomes for COVID-19 patients [6–9]. Additionally, this association is linked to a more severe clinical course and increased mortality rates from COVID-19. In addition to cardiovascular diseases, hypertension, and diabetes mellitus, chronic obstructive pulmonary disease is also a common comorbidity among COVID-19 patients [10, 11]. Individuals with COVID-19 and multiple comorbidities may exhibit an imbalanced immune response due to immune system dysfunction and chronic inflammation associated with the presence of multiple comorbidities. This dysfunctionality can result in a more severe disease outcome, increased risk of complications, and higher mortality due to direct or indirect modifications of the immune response caused by comorbidities [12–17]. Studies on the outcomes of these patients are rare, and data are very limited. Patients with chronic obstructive pulmonary disease (COPD) generally have increased susceptibility to viral infections, likely due to reduced production of interferon 1 (IFN1) or immunosenescence, characterized by an increased number of impaired T lymphocytes and memory T cells [18]. Some available studies suggest that the prevalence of acute kidney injury (AKI) among patients with COVID-19 is low, while the pathophysiological processes of acute kidney failure due to COVID-19 infection remain unknown [19]. There is increasing evidence that new chronic conditions may arise after acute COVID-19 illness. Data from US administrative claims showed that 14% of adult patients who had COVID-19 developed new clinical conditions within 6 months, a 1.65% higher incidence than after other viral infections. Clinical consequences included interstitial lung disease, respiratory insufficiency, congestive heart failure, arrhythmia, and type 2 diabetes [20]. The aim of this study is to determine which comorbidity has the greatest negative impact on disease worsening or is a poor prognostic comorbidity within COVID-19, as well as to understand the gender and age representation of the subjects and comorbidities.

## Participants and research methods

This retrospective study focuses on studying comorbidities in patients with confirmed SARS-CoV-2 infection using the RT-PCR method, who were hospitalized at the Cantonal Hospital Zenica. The study covered a three-month period (February, March, April) in 2021, during the third wave of the pandemic in Bosnia and Herzegovina, with the COVID-19 Multi Real-time RT-PCR kit used for virus confirmation. It is important to note that the vaccination status of the participants was not taken into account when designing the study. The data were analyzed with the approval of the Ethical Commission of the Cantonal Hospital Zenica, number: 00-03-35-2286-6/20, and the medical records of patients served as the data source. The analysis was conducted using the statistical package for social sciences SPSS, with regression analysis used as the method for data analysis. The aim of the research was to examine the impact of six comorbidities (hypertension, diabetes mellitus, cardiovascular diseases, urinary system diseases, thyroid diseases, and other comorbidities) as independent variables on the severity of COVID-19 illness as the dependent variable.

### Role of funding sources

This study has no sponsors who could have any role in study design, data collection, data analysis, interpretation, writing the final paper, or in the decision to submit the paper for publication.

## Results

The study involves a sample of 915 participants divided into three groups, as follows: the first group consists of deceased participants with 250 participants, the second group consists of participants on the ventilator with 151 participants, and the third group consists of recovered participants with 514 participants, as shown in Table 1.

**Table 1:** Participants by gender and age

Participants by group	Age groups	Gender		Total
		Male	Female	
Deceased	18 to 40 years	0 (00.0%)	1(100,0%)	1 (100,0%)
	41 to 64 years	17 (53.1%)	15 (46.9%)	32 (100,0%)
	65 and over	99 (45.6%)	118 (54.4%)	217 (100,0%)
	Total	116 (46.4%)	134 (53.6%)	250 (100,0%)
On a respirator	18 to 40 years	3 (50.0%)	3 (50.0%)	6 (100,0%)
	41 to 64 years	47 (65.3%)	25 (34.7%)	72 (100,0%)
	65 and over	47 (64.4%)	26 (35.6%)	73 (100,0%)
	Total	97 (64.2%)	54 (35.8%)	151 (100,0%)
Recovered	18 to 40 years	13 (54.2%)	11 (45.8%)	24 (100,0%)
	41 to 64 years	126 (55.8%)	100 (44.2%)	226 (100,0%)
	65 and over	146 (55.3%)	118 (44.7%)	264 (100,0%)
	Total	285 (55.4%)	229 (44.6%)	514 (100,0%)
Total	18 to 40 years	16 (51.6%)	15 (48.4%)	31 (100,0%)
	41 to 64 years	190 (57.6%)	140 (42.4%)	330 (100,0%)
	65 and over	292 (52.7%)	262 (47.3%)	554 (100,0%)
	Total	498 (54.4%)	417 (45.6%)	915 (100,0%)

No man aged 18 to 40 years had a fatal outcome, while only one woman in the same age group died. Among those aged 41 to 64 years, there were 17 (53.1%) male participants, while among those aged 65 and older, there were 118 (54.4%) female participants. In the group of participants on the ventilator, there was an equal number of male and female participants aged 18 to 40 years, while men in the age groups of 41 to 64 years and 65 and older outnumbered women by about 65%. In the group of recovered participants, the male gender dominated across all three age groups, with over 55%.

Table 2 shows the prevalence of comorbidities, with hypertension being the most prevalent at 53.7% among recovered participants. When examining the distribution of diabetes mellitus, Type 2 diabetes was the most prevalent among recovered participants at 54.2%. Among cardiovascular diseases, cardiomyopathies persisted the most, especially among recovered participants at 56.4%, while cardiomyopathies with atrial fibrillation persisted most among deceased participants at 59.6%. Triple-vessel or double-vessel coronary artery disease persisted somewhat less, especially among recovered participants at 68%, while the condition after myocardial infarction persisted most among deceased participants at 53.6%. Among urinary system diseases, nephropathy was most prevalent among recovered participants at 45.5%, while renal insufficiency was most prevalent among deceased participants at 53.6%. Hypothyroidism was widespread at 42.9% among recovered participants, as well as hyperthyroidism at 50%. Among other comorbidities, tumor formations persisted most among recovered participants at 55%. Anemia was more common among recovered participants at 52%, while chronic obstructive pulmonary disease was predominant in 53.8% within the same group of participants. Conditions post-intracranial bleeding were most common among deceased participants at 53.8%, while obesity was most common among recovered participants at 56.2%. The imbalance in sample sizes by gender in each comorbidity can potentially affect the accuracy and reliability of research results.

**Table 2:** Comorbidities by participant groups

Comorbidities		Deceased participants	Participants on ventilators	Recovered participants	Total
Participants		250 (27,3%)	151 (16,5%)	514 (56,2%)	915 (100,0%)
Hypertension		170 (31,1%)	83 (15,2%)	294 (53,7%)	547 (100%)
Diabetes mellitus type 2		90 (33,7%)	32 (12%)	145 (54,3%)	267 (100,0%)
Cardiovascular diseases	Cardiomyopathy	42 (35,9%)	9 (7,7%)	66 (56,4%)	117 (100,0%)
	Cardiomyopath/atrial fibrillation	28 (59,6%)	4 (8,5%)	15 (31,9%)	47 (100,0%)
	Three/two-vessel coronary disease	5 (20,0%)	3 (12,0%)	17 (68,0%)	25 (100,0%)
	AMI/st post MI	10 (47,6%)	7 (33,3%)	4 (19,0%)	21 (100,0%)
Urinary system diseases	Nephropathy	4 (36,4%)	2 (18,2%)	5 (45,5%)	11 (100,0%)
	Renal insufficiency	15 (53,6%)	5 (17,9%)	8 (28,6%)	28 (100,0%)
Thyroid gland diseases	Hypothyroidism	9 (21,4%)	15 (35,7%)	18 (42,9%)	42 (100,0%)
	Hyperthyroidism	3 (30,0%)	2 (20,0%)	5 (50,0%)	10 (100,0%)
Other comorbidities	Tumors	18 (45,0%)	-	22 (55,0%)	40 (100,0%)
	Anemia	10 (40,0%)	2 (8,0%)	13 (52,0%)	25 (100,0%)
	COPD	26 (33,3%)	10 (12,8%)	42 (53,8%)	78(100,0%)
	St post stroke	14 (53,8%)	5 (19,2%)	7 (26,9%)	26 (100,0%)
	Obesity	5 (31,3%)	4 (16,5%)	7 (56,2%)	16 (100,0%)

COPD-Chronic obstructive pulmonary disease

Table 3 shows that comorbidities are more common in older age groups, with almost all comorbidities being most prevalent in individuals aged over 65 years. An exception is obesity, which is equally prevalent in the age group of 41–64 years and in the age group of 65 years and above.

**Table 3:** Comorbidities by age groups of participants

Comorbidities		18 to 40 years	41 to 64 years	65 and over	Total
Hypertension		10 (1,8%)	172 (31,4%)	365(66,7%)	547 (100%)
Diabetes mellitus type 2		3 (1,1%)	82 (30,7%)	182 (68,2%)	267 (100,0%)
Cardiovascular diseases	Cardiomyopathy	-	33 (28,2%)	84 (71,8%)	117 (100,0%)
	Cardiomyopathy/atrial fibrillation	-	7 (14,9%)	40 (85,1%)	47 (100,0%)
	Three/two-vessel coronary disease	2 (8,0%)	10 (40,0%)	13 (52,0%)	25 (100,0%)
	AMI/st post MI	-	7 (33,3%)	14 (66,7%)	21 (100,0%)
Urinary system diseases	Nephropathy	-	3 (27,3%)	8 (72,7%)	10 (100,0%)
	Renal insufficiency	1 (3,6%)	9 (32,1%)	18 (64,3%)	28 (100,0%)
Thyroid gland diseases	Hypothyroidism	2 (4,8%)	14 (33,3%)	26 (61,9%)	42 (100,0%)
	Hyperthyroidism	2 (20,0%)	1 (10,0%)	7 (70,0%)	10 (100,0%)
Other comorbidities	Tumors	2 (5,0%)	15 (37,5%)	23 (57,5%)	40 (100,0%)
	Anemia	4 (16,0%)	10 (40,0%)	11 (44,4%)	25 (100,0%)
	Chronic obstructive pulmonary disease	1 (1,3%)	25 (32,1%)	52 (66,7%)	78 (100,0%)
	St post stroke	1 (3,8%)	4 (15,4%)	21 (80,8%)	26 (100,0%)
	Obesity	2 (12,5%)	7 (43,8%)	7 (43,8%)	16 (100,0%)

The analysis of the Pearson correlation coefficient in Table 4 shows that all predictor variables, such as diabetes mellitus, hypertension, cardiovascular diseases, urinary system diseases, and other comorbidities, are correlated with the criterion variable, which in this case is participants by groups, and the analyzed coefficients of hypertension, cardiovascular diseases, urinary system diseases, and other comorbidities are statistically significant. Specifically, the highest correlation between participants with different disease severity levels by groups as the criterion variable is with cardiovascular diseases as the predictor variable. This result suggests that there is a connection between the degree of illness intensity in participants and an increased risk of cardiovascular diseases. A lower coefficient compared to patients with different degrees of illness intensity was observed in relation to other comorbidities, urinary system diseases, and hypertension. This result indicates a variable degree of illness among participant groups, which may impact the occurrence of urinary system diseases, hypertension, and other comorbidities. The lowest coefficient was observed between different degrees of illness and diabetes mellitus, indicating a negative correlation. In other words, there is a reduced presence of diabetes mellitus in participants with different degrees of illness. Additionally, we wanted to determine the impact of all predictor variables on the severity of illness in all participants by groups, including deceased participants, participants on respirators, and recovered participants. The regression analysis model is presented in Table 5.

**Table 4:** Correlation analysis of participants by groups with comorbidities

Correlation	Participants by groups	Diabetes mellitus	Hypertension	Cardiovascular diseases	Urinary system diseases	Thyroid gland diseases	Other comorbidities
Participants by groups	1	-0,056	0,087**	0,106**	0,089**	0,021	0,095**
Diabetes mellitus		1	0,226**	0,071*	0,117**	0,005	-0,015
Hypertension			1	0,280*	0,082*	0,015	0,054
Cardiovascular diseases				1	0,099**	0,004	0,100**
Urinary system diseases					1	-0,029	0,032
Thyroid gland diseases						1	0,002
Other comorbidities							1

* Indicates that the correlation coefficient is significant at the 0.05 level

** Indicates that the correlation coefficient is significant at the 0.01 level

**Table 5:** Parameters of the analyzed model

Model Summary^b^				
Model	R	R Square	Adjusted R Square	Std. Error of the Estimate
1	0,369^a^	0,290	0,022	0,858

a. Predictors: (Constant), Other comorbiditties, Thyroid diseases, Diabetes mellitus, Cradiovascular diseases, Urinary system diseases, Hypertension

b. Dependent Variable: Participants by groups

Considering the model, it can be concluded that there is a low correlation between variables. The coefficient of determination is 0.290, which also represents the representativeness of the model, meaning that this model explains 29% of the variables, or participants who have developed some of the listed comorbidities, while other unknown factors influence the rest.

By calculating the ratio of mean square and residual mean, we obtain the empirical value of the F test. Based on the sample size and the empirical value of the F test, we get a significance value which, in the case of multiple regression, is 0.000. The ANOVA test in Table 6 examines the relationship between the criterion variable, which is patients grouped by the severity of the disease, and the predictor variables, which include diabetes mellitus, cardiovascular diseases, urinary system diseases, hypertension, thyroid diseases, and other comorbidities. The analysis showed significant compatibility between patient groups with different disease severities and the presence of the mentioned factors, indicating a statistically significant relationship between these variables. Based on this, it can be concluded that these variables have a high degree of dependence. Therefore, the assumption of correlation has been proven, meaning there is a significant connection between patients with different disease severities as the dependent variable and diabetes mellitus, cardiovascular diseases, urinary system diseases, hypertension, thyroid diseases, and other comorbidities as independent variables, and the coefficient of determination is significant because *p* = 0.000 < 0.05.

**Table 6:** Anova Test of Model Significance

ANOVA^a^						
Model		Sum of Squares	df	Mean Square	F	Sig.
1	Regression	19,682	6	3,280	4,458	0,000^b^
	Residual	668,147	908	0,736		
	Total	687,830	914			

a. Dependent Variable: Participants by groups

b. Predictors: (Constant), Other comorbiditties, Thyroid diseases, Diabetes mellitus, Cradiovascular diseases, Urinary system diseases, Hypertension

Based on the previous table, it can be concluded that the multiple regression model, or function, looks like this:

$$\begin{array}{l}{\rm{y = 0}}{\rm{.738 + 0}}{\rm{.059X1 + 0}}{\rm{.085X2 + 0}}{\rm{.045X3 }}\\{\rm{ + 0}}{\rm{.226X4 + 0}}{\rm{.044X5 + 0}}{\rm{.046X6}}\end{array}$$

where:

X1 - diabetes mellitus, X2 - hypertension, X3 - cardiovascular diseases, X4 - urinary system diseases, X5 - other comorbidities.

Based on the results of the analysis presented in Table 7, it can be concluded that the presence of urinary system diseases has the greatest negative impact on the worsening of Covid-19, and it should be noted that the tested beta constant is statistically significant because it is 0.030 < 0.05. Increased presence of cardiovascular diseases also affects the worsening of Covid-19, and the tested beta constant is statistically significant as it is 0.030 < 0.05. The presence of other comorbidities also influences the worsening of Covid-19, and the tested beta constant is statistically significant at 0.012 < 0.05. Arterial hypertension has a minor impact on the worsening of Covid-19, and the tested beta constant is not statistically significant at 0.169 > 0.05. The same applies to diabetes mellitus, which also has a minor impact on the worsening of Covid-19, and the tested beta constant is not statistically significant at 0.336 > 0.05, as well as thyroid diseases at 0.506 > 0.05. It can be concluded that an increase in the presence of urinary system diseases, cardiovascular diseases, and other comorbidities indicates a tendency towards a more severe degree of Covid-19 or poor prognostic comorbidities, whose coefficients are the highest and statistically significant since *p* < 0.05.

**Table 7:** Model coefficients

Coefficients^a^								
Model		Unstandardized Coefficients		Standardized Coefficients	*t*	Sig.	95,0% Confidence Interval for B	
		B	Std. Error	Beta			Lower Bound	Upper Bound
1	(Constant)	0,738	0,401		1,839	0,046	-0,050	1,526
	Diabetes mellitus	0,059	0,062	0,032	0,962	0,336	-0,062	0,181
	Hypertension	0,085	0,062	0,048	1,375	0,169	-0,036	0,206
	Cradiovascular diseases	0,045	0,021	0,075	2,173	0,030	0,004	0,085
	Urinary system diseases	0,226	0,104	0,072	2,170	0,030	0,022	0,431
	Thyroid diseases	0,044	0,066	0,022	0,665	0,506	-0,086	0,174
	Other comorbiditties	0,046	0,018	0,083	2,525	0,012	0,010	0,081

a. Dependent Variable: Participants by groups

## Discussion

Many similar studies have found a poorer outcome in older populations with specific comorbidities affected by COVID-19 [21]. Given the significant limitations in all studies, more detailed analyses and stratifications by comorbidities are needed to assess their impact on the outcome of COVID-19. In our study, the dominant age group was 65 years and older, as well as male gender. In our study, the dominant age group was 65 years and older, as well as male gender. The severity of COVID-19 in older people may be associated with immunosenescence, changes in cytokine patterns, activation of inflammatory pathways, and disrupted innate and adaptive immune responses, with aging remaining the most significant risk factor for mortality from COVID-19, while the prevalence of male gender is cited as a cause due to higher expression of ACE2 receptors [22, 23]. Various hypotheses have been proposed to explain gender differences in morbidity and mortality due to COVID-19, such as innate differences in the male and female immune systems, but this has not been proven. The most common comorbidity associated with COVID-19 is hypertension and diabetes mellitus, which have been reported as comorbidities due to disease progression and increased mortality from COVID-19 [24]. Recent data suggest that arterial hypertension does not appear to be as significant a risk factor for poor outcomes in COVID-19 as other cardiovascular diseases such as heart failure or coronary artery disease. These results are partly in line with our study, as arterial hypertension and diabetes mellitus were the most commonly reported comorbidities, where arterial hypertension had a strong association with COVID-19 but a weak impact on disease severity without statistical significance. Given that hypertension is prevalent in the general population and strongly associated with age, which is again a major risk factor for COVID-19, it is difficult to clearly and precisely determine the impact of one or the other [25]. Although the COVID-19 pandemic has affected all age groups, older adults are considered a particularly vulnerable population requiring greater protection due to the more severe health consequences of infection. It has been shown that individuals over 65 years of age account for 80% of hospitalizations and have a 23 times higher risk of death compared to those under 65 years of age [26]. In line with this, Puchongmarti et al. point out that COVID-19 patients between the ages of 70 to 79 have a mortality rate of up to 8%, while patients over 80 years of age have a rate of 15%. Patients with comorbid diseases such as cardiovascular diseases, chronic obstructive pulmonary disease, diabetes mellitus, cancer, and hypertension have a higher risk of severe illness and death, illustrating that comorbidities in the elderly population worsen the outcome of COVID-19. They conclude that important clinical factors associated with hospital mortality in older patients with COVID-19 include age, gender, diastolic blood pressure, body temperature, GCS score, total bilirubin, and CRP. These parameters can aid in triage and decision-making for this important patient population during times of limited resources during the COVID-19 pandemic [27]. Our research confirms that comorbidities are predominant in the older age group, where almost all comorbidities are most prevalent in the age group of 65 years and older. The only exception is obesity, which is equally prevalent in the age groups of 41 to 64 years and 65 years and older. As the number of comorbid conditions increases with age, this could be another logical explanation for the observed increase in mortality among older patients. While mortality from the disease is higher in older individuals with other conditions such as cardiovascular diseases, changes associated with immunosenescence may explain the increased susceptibility to infections and disproportionately high mortality from COVID-19 in older patients [28]. Recent observational, pathological, imaging, and clinical studies have clarified the short-term and long-term effects of COVID-19 on the cardiovascular system [29]. Numerous multinational studies show that older patients with cardiovascular diseases (CVD) are at high risk for adverse outcomes of COVID-19 due to the severity of the disease itself and myocardial injury, rather than direct myocardial injury caused by viral particles [30]. Furthermore, COVID-19 disease is associated with numerous cardiovascular (CV) complications, including arrhythmia, myocardial injury, cardiomyopathy, and thrombosis, leading to a “vicious cycle“ [13, 31, 32]. Although the exact pathophysiological mechanisms underlying the high-risk interaction between COVID-19 and existing cardiovascular disease are not fully elucidated, Drigin et al. postulate that the increased susceptibility to COVID-19 infection and cardiovascular complications associated with COVID-19 may be partially due to immune dysregulation and inflammation related to cardiovascular diseases exacerbated by viral infection [32]. Our study also supports their stance and suggests that cardiovascular diseases have a strong association and impact on the severity of COVID-19, leading to fatal outcomes. Previous studies on the involvement of the kidneys in patients with COVID-19 are contradictory. In initial studies and reports, the frequency of kidney diseases in patients with COVID-19 was negligible, and limited results were reported on the frequency of acute kidney injury in patients with COVID-19, while newer studies report a higher frequency of kidney abnormalities in patients with COVID-19 [33]. According to the results of some studies, kidney damage is commonly seen in conjunction with heart damage, likely because both of these complications are predictors of severe disease [34]. Although it is known that the Sars-CoV-2 virus primarily targets the lungs, the mechanisms leading to kidney dysfunction remain unclear. Mouliou et al. state that social vulnerability to respiratory infections is equally shaped in women and younger generations as in older individuals, with a noticeable association with previous lung inflammation and headaches, while no significant correlation has been observed with underlying medical conditions. Kidney disease is a major complication of viral infection, which can cause both acute and chronic kidney disease through various mechanisms, such as immune-mediated injury, direct injury by viral cells, systemic effects, and nephrotoxicity caused by antiviral drugs and hypoxia of renal tissue [35, 36]. Specific mechanisms for COVID-19 include the entry of SARS-CoV-2 into the kidneys and the binding of SARS-CoV-2 to the ACE2 receptor on the host cell membrane; in the kidneys, the ACE2 receptor is expressed in the apical borders of proximal tubules, as well as in podocytes [37]. It is known that COVID-19 stimulates unbalanced activation of the renin-angiotensin-aldosterone system (RAAS), causing regulation of ACE2 membrane-bound receptors that promote the accumulation of angiotensin II by reducing its degradation into angiotensin 1 [7]. Unbalanced activation of the RAAS leads to inflammation, vasoconstriction, and fibrosis at the level of the kidneys [38]. In addition, patients with comorbid chronic kidney disease (CKD) have a higher risk of upper respiratory tract infections and pneumonia due to persistent proinflammatory state and defects in innate and adaptive immunity [39]. While any medication can damage the kidneys, COVID-19 or anti-inflammatory drugs are not exceptions to this rule, and Cheng et al. posit that patients with COVID-19 have a high prevalence of kidney disease upon admission and a high rate of hospital mortality [40, 41]. Similarly, Fabrizi et al. conclude in their meta-analysis that there is a consistent relationship between the development of acute illnesses and poor outcomes (mortality rate) in hospitalized patients with COVID-19 [42]. These data are consistent with the results of our study, where we found a strong association and negative impact of urinary system diseases on the severity of COVID-19, which can lead to fatal outcomes.

## Conclusion

Our study has shown that comorbidities such as urinary system diseases, CVD, and other comorbidities tend to have a negative impact on COVID-19 disease, leading to a tendency towards poor outcomes that result in mortality, while diabetes mellitus and hypertension have an impact but without statistical significance. This study has limitations such as a retrospective design, and it is well-known that prospective studies with data at baseline and follow-up provide better evidence. Additionally, the study provides data from a single institution, suggesting the need for larger multicenter studies focusing on reporting comorbidities in admitted COVID-19 patients. Patients who were not hospitalized, and therefore were not included in the study, are in a significant number which could influence the study results. The research did not include the vaccination status of participants due to the lack of mandatory vaccinations during the course of the COVID-19 infection at the time of the study, as well as the limited availability of vaccines during that period which could affect the study results due to reported adverse events that in some cases could resemble COVID-19 symptoms. The study utilized a Multi Real-time RT-PCR kit which is a highly sensitive, specific, and rapid test for reliably detecting viral RNA in small samples with a low error rate, supporting the reliability of the data obtained in this study. Therefore, the study certainly makes a slight advancement in discovering data in the “vicious circle” of the impact of comorbidities on COVID-19 disease.

## Data Availability

The datasets generated and/or analyzed during the current study are not publicly available due to hospital policy that restricts the sharing of data. Our hospital has strict guidelines to protect patient privacy and medical confidentiality. However, access to the data can be requested by qualified researchers after obtaining appropriate permissions from the hospital and completing any necessary legal requirements.

## References

[1] Adab P, Haroon S, E O’Hara M, Jordan RE (2022). Comorbidities and Covid-19. BMJ.

[2] Mouliou DS, Pantazopoulos I, Gourgoulianis KI (2022). COVID-19 smart diagnosis in the Emergency Department: all-in in practice. Expert Rev Respir Med.

[3] Mouliou DS, Gourgoulianis KI (2021). False-positive and false-negative COVID-19 cases: respiratory prevention and management strategies, vaccination, and further perspectives. Expert Rev Respir Med.

[4] COVID 19 and Your Health. Centers for Disease Control and Prevention. 2021. https://www.cdc.gov/coronavirus/2019-ncov/need-extra-precautions/people-with-medical-conditions.html. [Last accessed on 2023 Mart 04].

[5] Elemam NM, Hannawi H, Salmi IA, Naeem KB, Alokaily F, Hannawi S (2021). Diabetes mellitus as a comorbidity in COVID-19 infection in the United Arab Emirates. Saudi Med J.

[6] Guan WJ, Ni ZY, Hu Y (2020). Clinical characteristics of coronavirus disease 2019 in China. N Engl J Med.

[7] Tadic M, Cuspidi C, Mancia G, Dell’Oro R, Grassi G (2020). COVID-19, hypertension and cardiovascular diseases: should we change the therapy?. Pharmacol Res.

[8] Samardžić J (2020). Covid-19 and antihypertensive therapy. Medicus.

[9] Guo T, Fan Y, Chen M, Wu X, Zhang L, He T, Wang H, Wan J, Wang X, Lu Z (2020). Cardiovascular implications of fatal outcomes of patients with Coronavirus Disease 2019 (COVID-19). JAMA Cardiol.

[10] Lian J, Jin X, Hao S, Cai H, Zhang S, Zheng L, Jia H, Hu J, Gao J, Zhang Y, Zhang X, Yu G, Wang X, Gu J, Ye C, Jin C, Lu Y, Yu X, Yu X, Ren Y, Qiu Y, Li L, Sheng J, Yang Y (2020). Analysis of epidemiological and clinical features in older patients with Coronavirus Disease 2019 (COVID-19) outside Wuhan. Clin Infect Dis.

[11] Li J, Wang X, Chen J, Zhang H, Deng A (2020). Association of renin-angiotensin system inhibitors with severity or risk of death in patients with hypertension hospitalized for Coronavirus Disease 2019 (COVID-19) infection in Wuhan, China. JAMA Cardiol.

[12] Gallo G, Calvez V, Savoia C (2022). Hypertension and COVID-19: current evidence and perspectives. High Blood Press Cardiovasc Prev.

[13] Clerkin KJ, Fried JA, Raikhelkar J, Sayer G (2020). Covid-19 and cardivasucular disease. Circulation.

[14] Ventura-Clapier R, Dworatzek E, Seeland U, Kararigas G, Arnal JF, Brunelleschi S, Carpenter TC, Erdmann J, Franconi F, Giannetta E, Glezerman M, Hofmann SM, Junien C, Katai M, Kublickiene K, König IR, Majdic G, Malorni W, Mieth C, Miller VM, Reynolds RM, Shimokawa H, Tannenbaum C (2017). D’Ursi AM, Regitz-Zagrosek V. Sex in basic research: concepts in the cardiovascular field. Cardiovasc Res.

[15] Deng YY, Zheng Y, Cai GY, Chen XM, Hong Q (2020). Single-cell RNA sequencing data suggest a role for angiotensin-converting enzyme 2 in kidney impairment in patients infected with 2019-nCoV. Chin Med J (Engl).

[16] Zou X, Chen K, Zou J, Han P, Hao J, Han Z (2020). Single-cell RNA-seq data analysis on the receptor ACE2 expression reveals the potential risk of different human organs vulnerable to 2019-nCoV infection. Front Med.

[17] Hallak J, Teixeira TA, Bernardes FS (2021). SARS-CoV-2 and its relationship with the genitourinary tract: implications for male reproductive health in the context of COVID-19 pandemic. Andrology.

[18] Halpin DMG, Criner GJ, Papi A, Singh D, Anzueto A, Martinez FJ, Agusti AA, Vogelmeier CF, Prevention of Chronic Obstructive Lung Disease. Global Initiative for the Diagnosis, Management, and. The 2020 GOLD Science Committee Report on COVID-19 and Chronic Obstructive Pulmonary Disease. Am J Respir Crit Care Med. 2021;203(1): 24–36. 10.1164/rccm.202009-3533SO PMC7781116 33146552

[19] Higham A, Mathioudakis A, Vestbo J, Singh D (2020). COVID-19 and COPD: a narrative review of the basic science and clinical outcomes. Eur Respir Rev.

[20] Daugherty SE, Guo Y, Heath K (2021). Risk of clinical sequelae after the acute phase of SARS-CoV-2 infection: retrospective cohort study. BMJ.

[21] Liu K, Chen Y, Lin R, Han K (2020). Clinical features of COVID-19 in elderly patients: a comparison with young and middle-aged patients. J Infect.

[22] Majdic G (2020). Could Sex/Gender differences in ACE2 expression in the lungs contribute to the large gender disparity in the morbidity and mortality of patients infected with the SARS-CoV-2 Virus?. Front Cell Infect Microbiol.

[23] Castelli EC, de Castro MV, Naslavsky MS, Scliar MO, Silva NSB, Pereira RN, Ciriaco VAO (2022). MUC22, HLA-A, and HLA-DOB variants and COVID-19 in resilient super-agers from Brazil. Front Immunol.

[24] Guo W, Li M, Dong Y, U Zhou H, Zhang Z, Tian C (2020). Diabetes is a risk factor for the progression and prognosis of COVID-19. Diabetes Metab Res Rev.

[25] Driggin E, Madhavan MV, Bikdeli B (2020). Cardiovascular considerations for patients, Health Care Workers, and Health systems during the COVID-19 pandemic. J Am Coll Cardiol.

[26] Mueller AL, McNamara MS, Sinclair DA (2020). Why does COVID-19 disproportionately affect older people?. Aging.

[27] Puchongmart C, Boonmee P, Jirathanavichai S, Phanprasert N, Thirawattanasoot N, Dorongthom T, Monsomboon A, Praphruetkit N, Ruangsomboon O (2023). Clinical factors associated with adverse clinical outcomes in elderly versus non-elderly COVID-19 emergency patients: a multi-center observational study. Int J Emerg Med.

[28] Yanez ND, Weiss NS, Romand JA, Treggiari MM (2020). COVID-19 mortality risk for older men and women. BMC Public Health.

[29] Chilazi M, Duffy EY, Thakkar A (2021). COVID and Cardicvascular Disease: what we know in 2021. CurrAtheroscler Rep.

[30] Xu J, Xiao W, Liang X, Shi L, Zhang P, Wang Y, Wang Y, Yang H (2021). A meta-analysis on the risk factors adjusted association between cardiovascular disease and COVID-19 severity. BMC Public Health.

[31] Bikdeli B, Madhavan MV, Jimenez D (2020). COVID-19 and thrombotic or thromboembolic disease: implications for Prevention, Antithrombotic Therapy, and Follow-Up: JACC Stateof-the-art review. J Am Coll Cardiol.

[32] Driggin E, Madhavan MV, Gupta A (2020). The role of vitamin D in cardiovascular disease and COVID-19. Rev EndocrMetabDisord.

[33] Gabarre P, Dumas G, Dupont T, Darmon M, Azoulay E, Zafrani L (2020). Acute kidney injury in critically ill patients with COVID-19. Intensiv. Care Med.

[34] Zhu J, Ji P, Pang J, Zhong Z, Li H, He C, Zhang J, Zhao C (2020). Clinical characteristics of 3062 COVID-19 patients: a meta-analysis. J Med Virol.

[35] Benedetti C, Waldman M, Zaza G, Riella LV, Cravedi P (2020). COVID-19 and the kidneys: an update. Front Med (Lausanne).

[36] Puelles VG, Lütgehetmann M, Lindenmeyer MT, Sperhake JP, Wong MN, Allweiss L (2020). Multiorgan and renal tropism of SARS-CoV-2. N Engl J Med.

[37] Chen A, Yin L, Lee K, He JC (2021). Similarities and differences between COVID-19-Associated Nephropathy and HIV-Associated Nephropathy. Kidney Dis (Basel).

[38] Vaduganathan M, Vardeny O, Michel T, McMurray J, Pfeffer M, Solomon S (2020). Renin-angiotensin aldosterone system inhibitors in patients with COVID-19. N Engl J Med.

[39] Singh J, Malik P, Patel N (2022). Kidney disease and COVID-19 disease severity-systematic review and meta-analysis. Clin Exp Med.

[40] Vahdat S (2022). Clinical profile, outcome and management of kidney disease in COVID-19 patients - a narrative review. Eur Rev Med Pharmacol Sci.

[41] Cheng Y, Luo R, Wang K, Zhang M, Wang Z, Dong L, Li J, Yao Y, Ge S, Xu G (2020). Kidney disease is associated with in-hospital death of patients with COVID-19. Kidney Int.

[42] Fabrizi F, Alfieri CM, Cerutti R, Lunghi G, Messa P (2020). COVID-19 and acute kidney Injury: a systematic review and Meta-analysis. Pathogens.

